# Vitamin D supplementation leading to hypervitaminosis D in a breastfed infant: A case report

**DOI:** 10.1002/ccr3.7635

**Published:** 2023-07-05

**Authors:** Sajal Twanabasu, Jeevan Ghimire, Sushan Homagain, Prabin Duwadee, Susmita Devkota, Ashish Bhandari, Prabhu Sunar

**Affiliations:** ^1^ Dhading Hospital Dhading Nepal

**Keywords:** breastfeeding, hypervitaminosis, infants, vitamin D

## Abstract

Breast milk alone is a poor and inadequate source of vitamin D. Many guidelines suggest supplementation of vitamin D to the newborns to prevent vitamin D deficiency. However, due to practices of outdoor breastfeeding and sunbathing, vitamin D supplementation may not be routinely required in our settings. Overzealous use of vitamin D supplementation and over‐the‐counter prescription may result in hypervitaminosis D.

## Graphical abstract

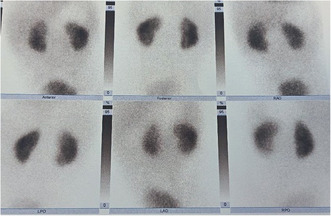

## 1 INTRODUCTION

Vitamin D is a fat‐soluble steroid hormone that plays a vital role in maintaining homeostasis of calcium and phosphorus. Ergocalciferol (vitamin D2) and cholecalciferol (vitamin D3) are obtained from various dietary plant and animal sources, respectively. Moreover, cholecalciferol (vitamin D3) is also obtained from 7‐dehydrocholesterol present in the skin via the UV rays. Vitamin D₂ and D₃ through various enzymatic reactions in liver and kidneys get converted into 1, 25‐dihydroxy vitamin D. Vitamin D helps in the absorption of the calcium and phosphorus from the gut, and it also stimulates the normal mineralization and growth of bones. The recommended daily allowance (RDA) for individuals aged 0–12 months is 10 mcg per day (10 mcg = 400 IU), whereas the RDA for individuals aged greater than 1 year is 15 mcg (15 mcg = 600 IU).^1^

Breast milk is a notoriously poor source of vitamin D.^2^ Exclusively breastfed infants not receiving supplemental vitamin D or adequate sunlight exposure are at increased risk of developing vitamin D deficiency and/or rickets.^3^ Hence, many guidelines suggest a daily supplementation of 400 IU in newborn babies.^4^, ^5^ However, large doses of vitamin D supplements lead to vitamin D toxicity. Vitamin D toxicity primarily present as elevated serum level of 25‐hydroxy vitamin D (i.e., >150 ng/mL), hypercalcemia, hyperphosphatemia, hypercalciuria, low parathyroid hormone, and high serum creatinine levels. Enhanced calcium absorption and bone resorption due to toxicity result in hypercalcemia, which can lead to deposition of calcium in many organs, particularly arteries and kidneys. We present a case of twin babies with hypervitaminosis resulting from prolonged intake of vitamin D in high doses.

## 2 CASE HISTORY

A 9‐month‐old male child was brought by his mother with complaints of excessive crying and decreased feeding for 2 days. There was no history of fever, cyanosis, noisy breathing, and chest retractions. She also gave a history of passing hard stool and frequent wet diapers. The child was conceived by in vitro fertilization and was first born among twins to a primigravida mother at 36 weeks of gestation with a birth weight of 2.05 kg. The other twin was female and had a birth weight of 2 kg. Postnatal period was uneventful. Both children were exclusively breastfed. General physical and systemic examinations were normal. Developmental milestones were appropriate for age. At the time of discharge, both twins were prescribed vitamin D supplements as many institutions are prescribing vitamin D in preterm breastfed infants as per American Academy of Pediatrics (AAP)/Indian Academy of Pediatrics (IAP) guidelines. Although the prescription for vitamin D was not available, on further evaluation, it was found that they were taking 800 IU of vitamin D daily from 1200 IU/mL dosage dropper with cumulative dose of more than 216,000 IU over 9 months.

**FIGURE 1:**
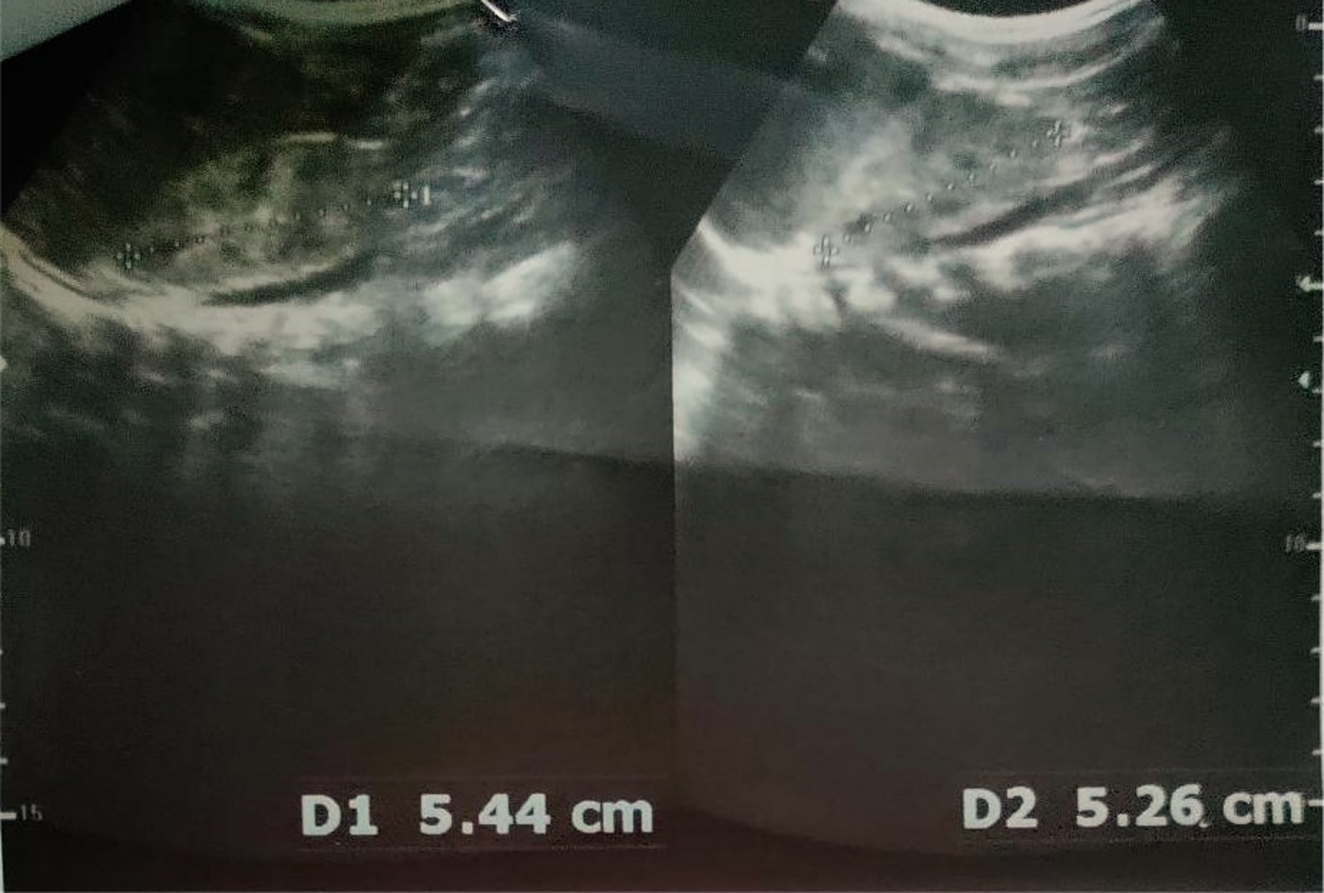
Right kidney.

**FIGURE 2:**
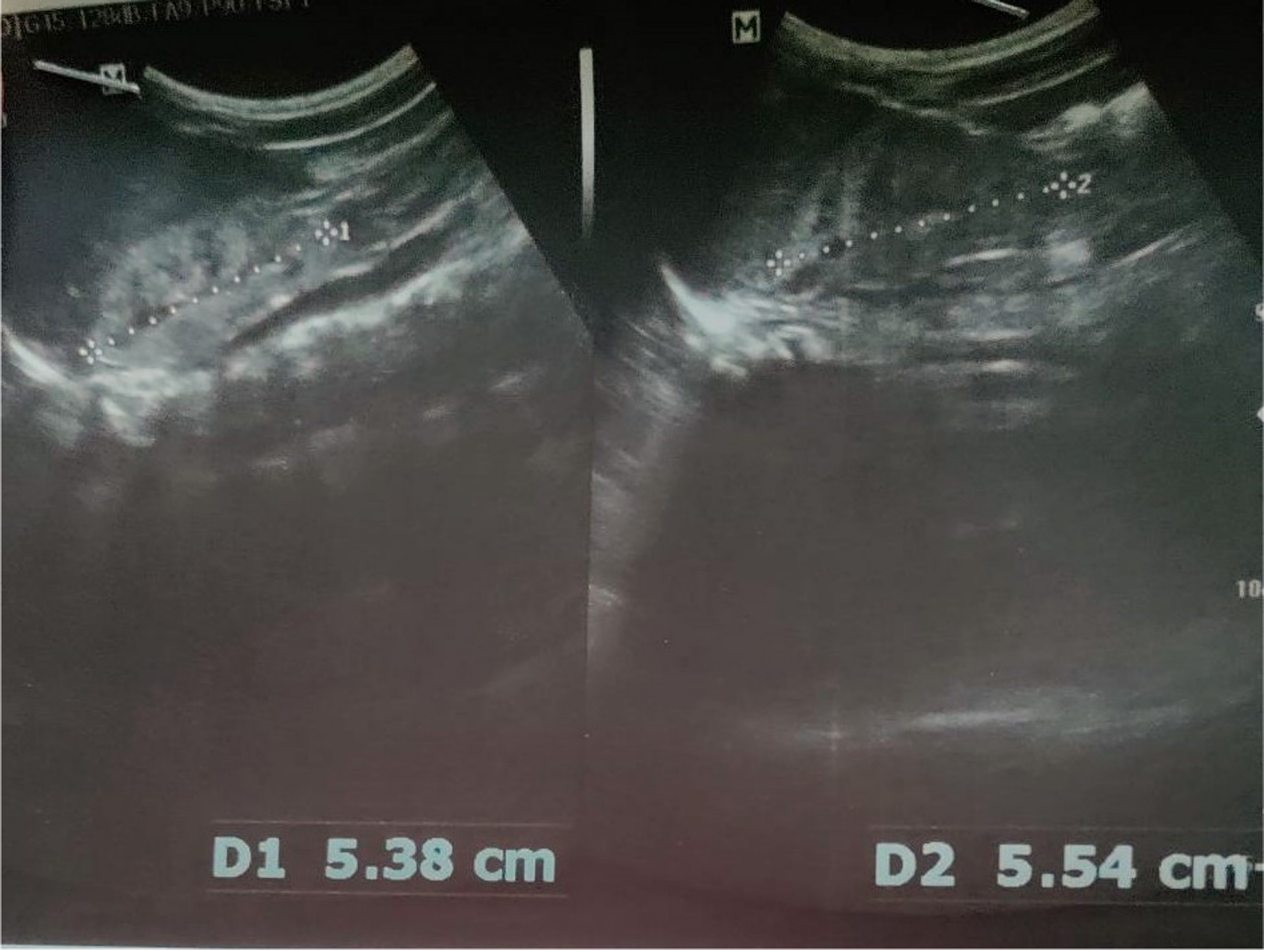
Left kidney.

**FIGURE 3:**
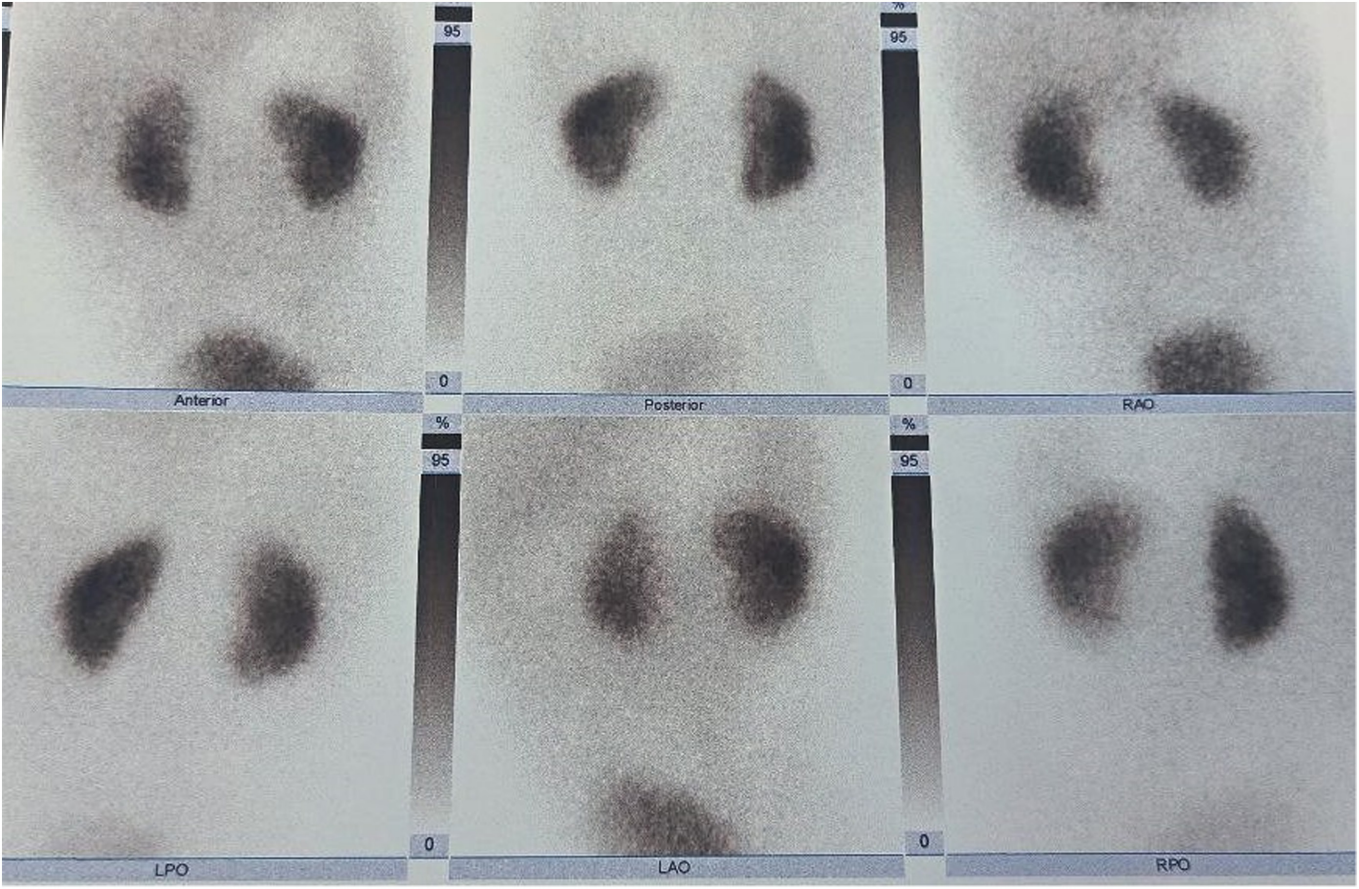
Dimercaptosuccinic acid (DMSA) scan.

Hypervitaminosis D was suspected, and investigations were sent which showed raised serum creatinine, hypercalcemia, high serum vit D (25 OH) level, normal phosphate, and low parathyroid hormone level. Patient was managed with IV hydration to increase renal excretion of calcium. Table 1 Ultrasonography (USG) abdomen showed bilateral renal parenchymal disease grade II (Figures 1 and 2) and (dimercaptosuccinic acid) DMSA was planned to check for renal scarring (Figure 3). Vitamin D supplementation was restricted. On the 10th day, serum creatinine normalized and the patient was discharged with advice to stop vitamin D drop, foods fortified with calcium and vitamin D, and follow‐up in 2 months.

**TABLE 1:** Laboratory parameters of the first twin.

Parameters	Admission	Discharge	Follow‐up (2 months)	Reference range
Vit D (ng/mL)	120	92	84.2	30–100
*Renal function test*				
Urea (mg/dL)	81	32	38	8–40
Creatinine (mg/dL)	2	0.8	0.7	0.2–0.8
Sodium (mEq/L)	138	140	145	130–147
Potassium (mEq/L)	4	3.8	5.4	3.4–5.6
Total calcium/	12.1/6	10.1/6	10.8	8.8–10.8
Phosphorous (mg/dL)				3.8–6.5
Ultrasound abdomen	Bilateral renal parenchymal disease grade II			
Urine routine	Normal			
Dimercaptosuccinic acid (DMSA) scan			Both normal size, preserved function, no cortical scar	
Twenty‐four‐hour urine Cr (μmol)	1233			8840–13,260
Twenty‐four‐hour urine Ca (μmol)	2.68			2.5–7.5
iPTH (pg/mL)	10.9			10–55

Although both twins were taking the same doses of vitamin D for the same period of time, the second twin had no symptoms. She was also evaluated. However, her laboratory investigations were within normal limits (Table 2) She was also advised to stop the vitamin D supplementation.

**TABLE 2:** Laboratory parameters of the second twin.

Parameters	Values	Reference range
Vit D (ng/mL)	92.5	30–100
*Renal function test*		
Urea (mg/dL)	34	8–40
Creatinine (mg/dL)	0.5	0.2–0.8
Sodium (mEq/L)	145	130–147
Potassium (mEq/L)	5.2	3.4–5.6
Calcium/	9.1/6.10	8.8–10.8
Phosphorous (mg/dL)		3.8–6.5
Ultrasound abdomen	Normal scan	
Urine RE/ME	Normal	
Twenty‐four‐hour urine Cr (μmol)	1095	8840–13,260
Twenty‐four‐hour urine Ca (μmol)	0.80	2.5–7.5

At 2‐month follow‐up, repeated investigations were normal. A DMSA scan was done which showed normal size kidneys with normal function and no cortical scarring (Figure 3).

## 3 DISCUSSION

Vitamin D, a fat‐soluble vitamin synthesized in the human body via exposure to the sun, is frequently used as both prescription and over‐the‐counter formulation. The rare cases of vitamin D toxicity are usually secondary to misuse of over‐the‐counter supplements, erroneous prescriptions.^6^

The high doses ingested are stored in liver and adipose tissues and saturate vitamin D receptors. Although there is negative feedback regulation by an enzyme 25 hydroxylase in the liver, it is not inadequate to prevent the buildup of vitamin D metabolites such as 25(OH)D, 24,25(OH)2D, 25,26(OH)2D, and 25(OH)D‐26,23‐lactone, leading to abnormal calcium homeostasis.^7^ The level of 25(OH) D correlates with vitamin D toxicity and is a marker of vitamin D stores. The level of 1,25(OH)2D however may be increased or normal as it is also regulated by parathyroid hormone and calcium.^8^

An average vitamin D content of human breast is around 22 IU/L that too in a vitamin D‐sufficient mother.^2^ So an infant receiving around 1 L of breast milk per day receives around 22 IU of vitamin D per day, which is way below the recommended daily dose of 400 IU per day. Hence, the American Academy of Pediatrics (AAP) recommends that all infants less than 1 year old should have a minimum intake of 400 IU of vitamin D per day beginning soon after birth including preterms.^5^ Similar is the recommendation from the Indian Academy of Pediatrics (IAP).^4^ The upper limit intake (the level above which there is risk of adverse events) for vitamin D supplementation is 1000 IU/d for infants of 0–6 months and 1500 IU/day for infants of 6–12 months.^9^ However it is just an adequate intake reference value, as RDAs have not been established for infants and it is found that standard total intake of 400 IU daily will achieve an average value of serum 25(OH)D above 30 ng/mL, with some infants of vitamin D deficient mothers taking longer to reach this value.^9^ There is however no evidence of clinical benefit of routinely giving higher doses.^10^ In one of the randomized controlled trials, even 250 IU of vitamin D3 was found sufficient among breastfed infants even in winter.^11^ Our patient received an excess of 800 IU per day over the recommended dose which resulted in hypervitaminosis. This excess might have resulted from over‐the‐counter prescription error, instead of 400 IU of Vitamin D dropper the baby was prescribed 800 IU of Vitamin D dropper. Interestingly though, despite receiving the similar dose of vitamin D, the other twin did not develop hypervitaminosis. There seems no plausible explanation for this, unless the caregiver administered different doses knowingly/unknowingly or the twin is tolerant.

In a study conducted in Nepal, vitamin D insufficiency [25(OH)D < 50 nmol/L] and deficiency (<30 nmol/L) prevalence were 3.6% and 0.6%, respectively, among newborn, in contrast to 59.8% and 14.0% among their mothers. The infants had adequate vitamin D status despite the mother being deficient.^12^ This situation can partly be explained by the practice of sunbathing the newborn after oil massages and outdoor breastfeeding, which is prevalent among Nepalese society.^12^ Although it needs to be validated through study of large sample size, this study showed that routine and robust vitamin D supplementation may not be required among newborn in our setting. Although vitamin D supplementation is not recommended in Nepal,^13^ we often follow the guidelines from IAP and AAP, so it is a routine practice in our setting to prescribe vitamin D supplementation to the newborns. This overzealous use of vitamin D due to fear of rickets and practice of following western guidelines might lead to increasing cases of hypervitaminosis.

Vitamin D toxicity in an infant presents with nonspecific and subtle symptoms such as poor feeding, constipation, intolerance to food, polyuria, dehydration, lethargy failure to thrive, emesis, and even diarrhea. are direct effects of hypercalcemia, and frequently correlate with the calcium level. Our patient had constipation, irritability, and frequent diaper wetting. The vasoconstrictive effect of calcium may have hypertension in some patients, and hypercalciuria may lead to nephrolithiasis and nephrocalcinosis.^14^, ^15^, ^16^, ^17^, ^18^ In a retrospective review of seven children (age 7.5–25 months) who were taking high doses (900,000–4000,000 U) over a period of 2–8 weeks, Joshi et al.^14^ mentioned similar symptoms in children along with hypercalcemia.

The goal of treatment is to rehydrate the patient as hypercalcemia causes dehydration and decreases the calcium level in the body. Infusion of normal saline (10–20 mL/kg) can be used to expand the extracellular compartment. Before adding diuretics to increase urinary excretion of calcium, adequate urine flow must be established to prevent precipitation of calcium leading to dehydration and nephrolithiasis. The continual intestinal calcium absorption may lead to rise in calcium level despite treatment. Such cases can be managed by adding prednisolone to treatment regimen as it lowers calcium absorption by decreasing 1‐α‐hydroxylation of 25(OH)D to 1,25(OH)2D in the kidney. Other interventions include use of calcitonin, bisphosphonates, and pamidronate in resistant cases.^19^, ^20^

## 4 CONCLUSION

Overzealous use of vitamin D supplementation in newborns for prolonged duration increases the risk of hypervitaminosis. Although the guidelines suggest the use of vitamin D in newborns, the prevalence of vitamin D deficiency, practices of breastfeeding, and other sociocultural practices in the local settings should be taken into account before prescribing it.

## AUTHOR CONTRIBUTIONS

**Sajal Twanabasu:** Conceptualization; writing – review and editing. **Jeevan Ghimire:** Writing – original draft; writing – review and editing. **Sushan Homagain:** Writing – original draft; writing – review and editing. **Prabin Duwadee:** Writing – original draft; writing – review and editing. **Susmita Devkota:** Writing – review and editing. **Ashish Bhandari:** Writing – review and editing. **Prabhu Sunar:** Writing – review and editing.

## FUNDING INFORMATION

None.

## CONFLICT OF INTEREST STATEMENT

None.

## PATIENT CONSENT STATEMENT

Written informed consent was obtained from the parents to publish this report in accordance with the journal's patient consent policy.

## Data Availability

Data will be provided by the corresponding author upon reasonable request.
